# Optimizing Liposomal Cisplatin Efficacy through Membrane Composition Manipulations

**DOI:** 10.1155/2011/213848

**Published:** 2011-01-24

**Authors:** Natalia Zisman, Nancy Dos Santos, Sharon Johnstone, Alan Tsang, David Bermudes, Lawrence Mayer, Paul Tardi

**Affiliations:** Department of Preformulation, Celator Pharmaceuticals Corp., 1779 West 75th Avenue, Vancouver, BC, Canada V6P 6P2

## Abstract

The first liposomal formulation of cisplatin to be evaluated clinically was SPI-077. Although the formulation demonstrated enhanced cisplatin tumor accumulation in preclinical models it did not enhance clinical efficacy, possibly due to limited cisplatin release from the formulation localized within the tumor. We have examined a series of liposomal formulations to address the in vivo relationship between cisplatin release rate and formulation efficacy in the P388 murine leukemia model. The base formulation of phosphatidylcholine: phosphatidylglycerol: cholesterol was altered in the C18 and C16 phospholipid content to influence membrane fluidity and thereby impacting drug circulation lifetime and drug retention. Phase transition temperatures (*T_m_*) ranged from 42–55°C. The high *T_m_* formulations demonstrated enhanced drug retention properties accompanied by low antitumor activity while the lowest *T_m_* formulations released the drug too rapidly in the plasma, limiting drug delivery to the tumor which also resulted in low antitumor activity. A formulation composed of DSPC : DPPC : DSPG : Chol; (35 : 35 : 20 : 10) with an intermediate drug release rate and a cisplatin plasma half-life of 8.3 hours showed the greatest antitumor activity. This manuscript highlights the critical role that drug release rates play in the design of an optimized drug delivery vehicle.

## 1. Introduction

Cisplatin or cis-diamminedichloroplatinum (II) belongs to a family of platinum-containing complexes that are used clinically to treat cancer and include carboplatin, oxaliplatin, and nedaplatin. Cisplatin is a widely used cytotoxic agent approved in the treatment of bladder, ovarian, testicular, cervical, head and neck, and nonsmall cell lung cancer [1–3]. Cisplatin was first documented in 1847 by M. Peyrone, and it was approved by FDA for a variety of cancers in 1978. The mode of action of cisplatin depends on the rate of hydrolysis of the chloride groups of the complex. Upon entering the cell, the neutral cisplatin molecule undergoes hydrolysis, in which a chlorine ligand is replaced by a molecule of water (termed aquation), generating a positively charged species. The resulting hydrolysis product is believed to be the active species, interacting with nucleophilic molecules including DNA [4], RNA, and protein. More specifically, the interactions result in interstrand and intrastrand cross-links that effectively halt DNA, RNA, and protein synthesis leading to the activation of the apoptotic cascade [5, 6]. Although cisplatin is a highly effective anticancer agent, it is noted for severe side effects including renal toxicity, gastrointestinal toxicity, nephrotoxicity, ototoxicity, and optic neuropathy which limits its use in the clinic [7–10].

Drug delivery vehicles, such as liposomes, have been employed to reduce the dose-limiting nephrotoxicity of cisplatin [10, 11]. Currently, there are no FDA-approved liposomal cisplatin formulations available; however, SPI-077 and Lipoplatin formulations have been tested in clinical trials [12–14]. SPI-077 is a sterically stabilized liposomal cisplatin formulation containing hydrogenated soy phosphatidylcholine, cholesterol, and methoxypolyethylene glycol (mPEG-DSPE) at a 51 : 44 : 5 molar ratio [12, 15, 16]. Although the formulation exhibited prolonged circulation in blood and was very well tolerated [16], it had very limited therapeutic activity in a Phase II clinical study in NSCLC, with an overall response rate of 4.5% [12]. The release of platinum from the SPI-077 formulation at the tumor site was subsequently investigated by Zamboni et al. [17] using microdialysis. In this study, B16 melanoma tumor homogenates were assayed for platinum accumulation following intravenous administration of SPI-077 or cisplatin. In the case of SPI-077-treated mice, approximately 4-fold higher platinum levels were associated with the tumors than that of cisplatin. However, analysis of tumor extracellular fluid by microdialysis revealed no detectable levels of unbound platinum in SPI-077-treated mice indicating very little release of cisplatin from the liposomes within the tumor. As a measure of cisplatin activity within the tumor, platinum DNA adduct analysis revealed approximately 4-fold lower adduct formation with SPI-077 compared to cisplatin. These results indicate that the poor clinical efficacy observed with SPI-077 is likely the result of low cisplatin release from the liposomes at the tumor site. Taken together, enhanced platinum delivery to the tumor site through improved lipid retention does not lead to improved therapeutic efficacy, due to decreased tumor exposure to free agent. 

Lipoplatin, another liposomal cisplatin formulation, is composed of dipalmitoyl phosphatidyl glycerol (DPPG), soy phosphatidyl choline, cholesterol, and mPEG-DSPE [13, 14, 18, 19]. It is currently undergoing a number of Phase II and Phase III clinical trials in combination with other cytotoxic agents such as gemcitabine, 5-fluorouracil, and vinorelbine, where lipoplatin is demonstrating substantially reduced renal toxicity, peripheral neuropathy, ototoxicity, and myelotoxicity [14, 19]. As with SPI-077, enhanced retention of cisplatin may improve tolerability of the liposomal cisplatin formulation but may also result in decreased antitumor activity.

To determine if the drug release rate plays a critical role in the overall therapeutic activity of a cisplatin formulation, a series of liposomes were prepared. The base formulation chosen for this study was composed of phosphatidylcholine (PC), phosphatidylglycerol (PG), and cholesterol (Chol) at a 7 : 2 : 1 mole ratio since this composition was previously used to control the release rates of irinotecan, floxuridine, cytarabine, and daunorubicin [20, 21]. Through small changes in the phospholipid acyl chain composition, we were able to generate a panel of cisplatin formulations with different release rates without changing the phospholipid molar ratio of the formulation. As the percentage of dipalmitoyl acyl chains increased and distearoyl decreased, there was a corresponding decrease in the thermal melting point of the formulation and an enhancement in cisplatin release rate in vivo. This series of liposomal formulations was evaluated for therapeutic activity in the P388 murine lymphocytic leukemia model. Based on the efficacy results, an optimal cisplatin release rate was identified. 

## 2. Materials and Methods

### 2.1. Reagents

1,2-distearoyl-*sn*-glycero-3-phosphatidylcho-line (DSPC), 1,2-dipalmitoyl-*sn*-glycero-3-phosphatidylcho-line (DPPC), and 1,2-distearoyl-*sn*-glycero-3-phosphatidy-lglycerol (DSPG) were purchased from Lipoid GMBH (Ludwigshafen, Germany). Cholesterol (CHOL) was obtained from Solvay Pharmaceuticals BV (Weesp, Netherlands). ^3^H-cholesteryl hexadecyl ether (CHE) was purchased from Perkin-Elmer Life Sciences (Woodbridge Ontario, Canada). Cisplatin lyophilized powder was purchased from PolyMed Therapeutics, Inc. (Houston, Texas, USA). All other chemicals were purchased from Sigma Chemical Co. (St. Louis, MO, USA).

### 2.2. Liposome Preparation

All liposome formulations were prepared by the extrusion technique. Briefly, the lipids were dissolved in chloroform/methanol/water (5 : 1 : 0.1, v/v/v) and mixed together at the indicated molar ratio. ^3^H-CHE was added to the formulations as a nonexchangeable, nonmetabolized lipid marker. The solvent mixture was evaporated under a stream of nitrogen gas and the sample was placed under high vacuum for 3 hours. The lipid films were rehydrated in 150 mM saline by gentle mixing at 70°C. The newly formed multilamellar vesicles (MLVs) were passed 2 times through an extruding apparatus (Northern Lipids Inc., Vancouver, BC, Canada) containing two stacked 200 nm and 8 times through 100 nm polycarbonate filters (Northern Lipids Inc., Vancouver, BC, Canada). The extrusion was carried out at 70°C. The mean diameter and size distribution of each liposome preparation were analyzed by quasielastic light scattering NICOMP model 380 submicron particle sizer (Pacific Scientific, Santa Barbara, CA, USA), and was typically 100 ± 20 nm by volume weighting. Cisplatin was passively loaded into extruded liposomes as previously described [22]. Briefly, a final solution containing 20 mg/ml lipid, 7.5 mg/ml cisplatin, and 7.5% ethanol was mixed and heated for 1 hour at 60°C. The ethanol and the unencapsulated cisplatin were removed by tangential flow filtration through polysulfone hollow fiber columns (500 kD, GE Healthcare) against 10 wash volumes of 150 mM saline. In the case of the DSPC/Chol formulation, however, cisplatin could not be efficiently encapsulated into the formulations after extrusion, presumably due to the high cholesterol content of the formulation. As a result, cisplatin was incorporated in this formulation by extruding in the presence of the drug. Although this formulation was included in the pharmacokinetic studies, it was not included in the efficacy due to concerns related to the stability of cisplatin following high temperature and pressure exposure. Differential scanning calorimetry (DSC) measurements were performed using a VP-DSC Microcalorimeter (MicroCal, Northampton, MA) with a scan rate of 60°C/hr. Liposomes were diluted to 5–20 mg/ml in saline, and the reference cell was filled with degassed saline. Buffer baselines were subtracted from the liposome scans, and the main gel to liquid-crystalline phase transition temperature was determined and recorded as the (*T*_*m*_) of the formulation. Data were analyzed using ORIGIN software.

### 2.3. Pharmacokinetic Analysis and Biodistribution

All animal experiments were conducted under the guidance of the UBC animal care committee. BDF-1 mice (3 mice per group) were injected with 3 mg/kg liposomal cisplatin into the lateral tail vein. At various time points, up to 48 hours after drug administration, blood was collected by cardiac puncture and collected into EDTA-containing microtainer tubes, cooled on ice and centrifuged at 900 × g for 10 min at 4°C. Platinum levels were analyzed by atomic absorption spectroscopy (Varian SpectrAA-240 Zeeman Graphite Furnace atomic absorption spectrometer; Varian, Canada Inc. ST-Laurent, Quebec, Canada), and lipid concentration was determined by quantifying tracer quantities of ^3^H-CHE by liquid scintillation counting (LS 6500 Multi Purpose Scintillation Counter; Beckman Coulter Canada Inc., Mississauga, Ontario, Canada). The plasma elimination data was modeled by PK Solutions (Summit Research Services, Montrose, Colorado, USA) to calculate pharmacokinetic parameters including half-life (*T*_1/2_) and area-under-the-moment curve (AUMC_0-tlast_).

For biodistribution studies, spleen, liver, and kidneys of the injected mice (3 mice per group) were collected up to 96 hours after drug administration. The tissues were excised into preweighed tubes and stored at −80°C. For analysis of total platinum, tissue samples were thawed and homogenized in 0.9% sterile saline (tissue: 0.9% NaCl at 1 : 5 w/v). Homogenate (100 **μ**l) was added to 100 **μ**l of 70% nitric acid and incubated at 70°C for 2 hours. The samples were cooled, diluted with 1% nitric acid and analyzed by graphite furnace atomic absorption spectrometer (AA240Z, Varian, Mulgrave, Victoria, Australia) containing a GTA 120 Zeeman graphite tube atomizer. Pt absorbance was measured at a wavelength of 265.9 nm.

### 2.4. In Vivo Efficacy Studies and Tumor Accumulation Studies

Efficacy studies were conducted in female BDF-1 mice (8 mice per group) injected i.p. with 500 **μ**l of 1 × 10^6^ P388 murine lymphocytic leukemia cells which were obtained from the National Cancer Institute tissue repository (Bethesda, Maryland, USA) and propagated in vivo as described previously [23]. Intravenous liposome administration commenced 24 hours after tumor cell inoculation. Mice were monitored daily for signs of stress and toxicity. Animals that showed signs of illness due to tumor progression were terminated, and the day of death was recorded as the following day. The treatment (T) and control (C) group lifespan were determined the day in which the last mouse in the group died. The percent increased lifespan (% ILS) was calculated as (T−C)/C.

## 3. Results and Discussion

### 3.1. Cisplatin Plasma Elimination as a Function of Lipid Composition

**Figure 1:**
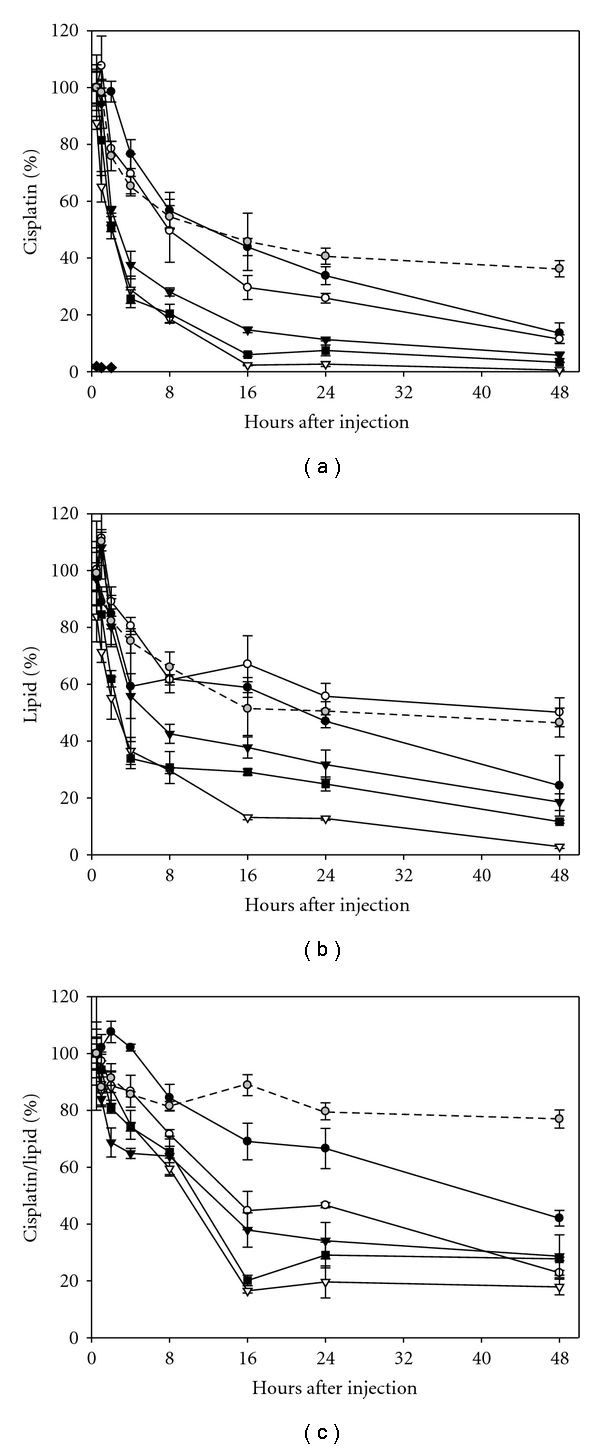
Percent of injected dose for cisplatin (a), lipid (b) and percent initial cisplatin/lipid ratio (c) in plasma following i.v. administration of cisplatin containing liposomes into BDF-1 mice. The formulation compositions: DSPC : DSPG : CHOL (7 : 2 : 1) (●); DSPC : DPPC : DSPG : CHOL (6 : 1 : 2 : 1) (∘); DSPC : DPPC : DSPG : CHOL (35 : 35 : 20 : 10) (▾); DSPC : DPPC : DPPG : CHOL (35 : 35 : 20 : 10) (■), DPPC : DPPG : CHOL (7 : 2 : 1) (▿); DSPC : CHOL (55 : 45) (gray circle); and free cisplatin (♦), were all administered at a cisplatin dose of 3 mg/kg (*n* = 3, Mean ± SD). Mouse plasma was collected at 0.5, 1, 2, 4, 8, 16, 24, and 48 hours after administration. The platinum concentration was determined by atomic absorption spectroscopy and the lipid by scintillation counting.

We evaluated the effect of different membrane compositions on the drug release properties of liposomes composed of phosphatidylcholine (PC), phosphatidylglycerol (PG), and cholesterol. The compositions were varied by evaluating 1,2-distearoyl (DS) or 1,-2-dipalmitoyl (DP) versions of PC and PG combined at different ratios while the cholesterol content was fixed at 10 mol%. The increase in molar % DS content in membrane composition of these low-cholesterol liposomes corresponded to an increase in membrane transition temperature (*T*_*m*_) (Table 1). All formulations showed a single transition temperature peak by DSC indicating good lipid mixing in the bilayer and an absence of phospholipid domain formation. Cisplatin was loaded into the formulations using a postextrusion method [22] to avoid exposing the drug to high temperatures and pressures required during liposome formation. The clearance profiles of the formulations were compared to conventional DSPC : Chol (55 : 45 mol%) liposomes, known for their slow clearance and enhanced drug retention properties. As can be seen from the plasma elimination of cisplatin (Figure 1(a)), the highest circulating plasma levels were observed with the conventional DSPC : Chol formulation where levels approaching 50% of the injected dose were still observed at 24 and 48 hours after administration. As previously predicted, the DSPC/Chol formulation showed very little change in the cisplatin-to-lipid ratio over 48 hours (Figure 1(c)) indicating that only a small percentage of cisplatin is released from this formulation in vivo.

**Figure 2:**
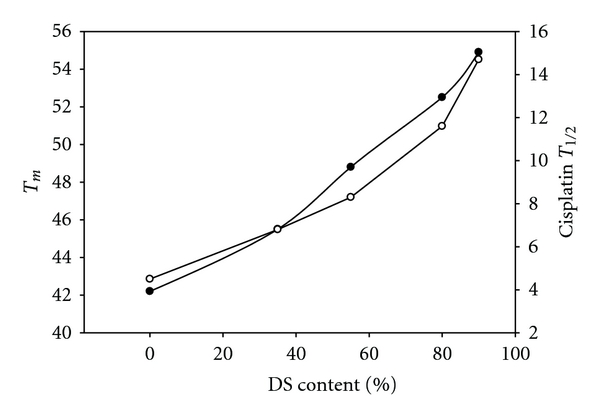
The effect of % DS content on the transition temperature (*T*_*m*_) of liposomal carrier (●) and on cisplatin half-life (∘) in the plasma.

For the low-cholesterol formulations a decrease in molar % DS corresponded to lower cisplatin levels in plasma. (Figure 1(a)). From these data various pharmacokinetic parameters were calculated including cisplatin half-life, cisplatin AUC, lipid half-life, and lipid AUC (Table 1). The pharmacokinetic results revealed a general trend where increasing DP content resulted in decreased cisplatin and lipid half-lives. The decreased lipid half-life may be the result of more fluid liposomal membranes binding higher amounts of serum protein. This has been shown previously to enhance liposomal clearance. To determine if the decreasing plasma cisplatin levels were simply the result of enhanced liposome clearance, changes in the drug-to-lipid ratio were calculated and plotted (Figure 1(c)). Based on these composite results, there is a clear difference in the drug retention properties of the various formulations. As with the cisplatin and lipid clearance, an increase in the DP content resulted in enhanced drug leakage. The effect of lipid composition (percent DS content) on the *T*_*m*_ of the liposomal carrier and on cisplatin half-life was plotted in Figure 2. This figure demonstrates a clear relationship between the membrane composition and platinum plasma levels. Although the set of five low-cholesterol formulations showed only a 12.7°C range (54.9–42.2°C) in transition temperatures, large effects were observed in the pharmacokinetic properties of these formulations. When comparing the most divergent formulations DSPC/DSPG/Chol (7 : 2 : 1, 90% DS) versus DPPC/DPPC/Chol (7 : 2 : 1, 90% DP), the DS based formulation circulated for 3-fold longer, and enhanced cisplatin circulation by 3.26-fold, resulting in a 16.3-fold improvement in cisplatin AUC. Even more surprising was the observation that formulations with moderately different lipid clearance profiles and transition temperatures could have dramatically different release rates. For example, when comparing DSPC/DSPG/Chol (7 : 2 : 1, 90% DS) to a blended formulation of DSPC : DPPC : DSPG : Chol (35 : 35 : 20 : 10, 55% DS), the cisplatin half-life is 1.8-fold faster in the blended composition which decreased the cisplatin AUC by 4.2-fold. These results highlight how small changes in the membrane transition temperature can have a dramatic effect on in vivo pharmacokinetics.

### 3.2. Impact of Cisplatin Release Rate on P388 Efficacy

**Figure 3:**
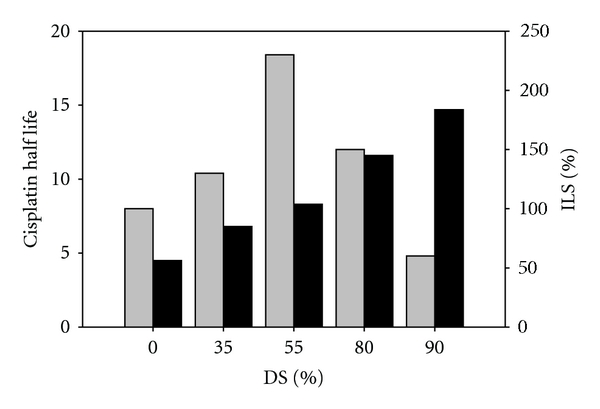
Cisplatin half-life (■) and the corresponding percent of increased lifespan (%ILS) (gray square) from the P388 murine lymphocytic leukemia model are plotted as a function of the liposomal membrane composition. Mice (*n* = 8) bearing P388 tumors received intravenous injections of liposomal cisplatin formulations at MTD doses on a Q4D × 3 dosing schedule.

All of the low-cholesterol formulations were evaluated for efficacy in the P388 murine lymphocytic leukemia model to determine if the different pharmacokinetic properties of the formulations would have an impact on the therapeutic activity of cisplatin. We compared the overall survival rates for each formulation to saline and calculated the percentage increased life span or %ILS. In Figure 3 the %ILS for each formulation is plotted along with its respective cisplatin release half-life. It is clear that enhancing cisplatin circulation lifetime did not result in improved cisplatin efficacy in the P388 model. In fact, the formulation with the longest cisplatin half-life (DSPC : DSPG:Chol 7 : 2 : 1 at 14.7 hours), has lowest %ILS compared to a formulation with an intermediate half-life (DSPC : DPPC : DSPG : Chol; 35 : 35 : 20 : 10 at 8.3 hours) and the highest %ILS. The second lowest %ILS values were observed for DPPC : DPPG : Chol which had the shortest cisplatin half-life of 4.5 hours. Taken together, high and low cisplatin release rates translated into poor biological performance.

### 3.3. Biodistribution of Liposomal Cisplatin

**Figure 4:**
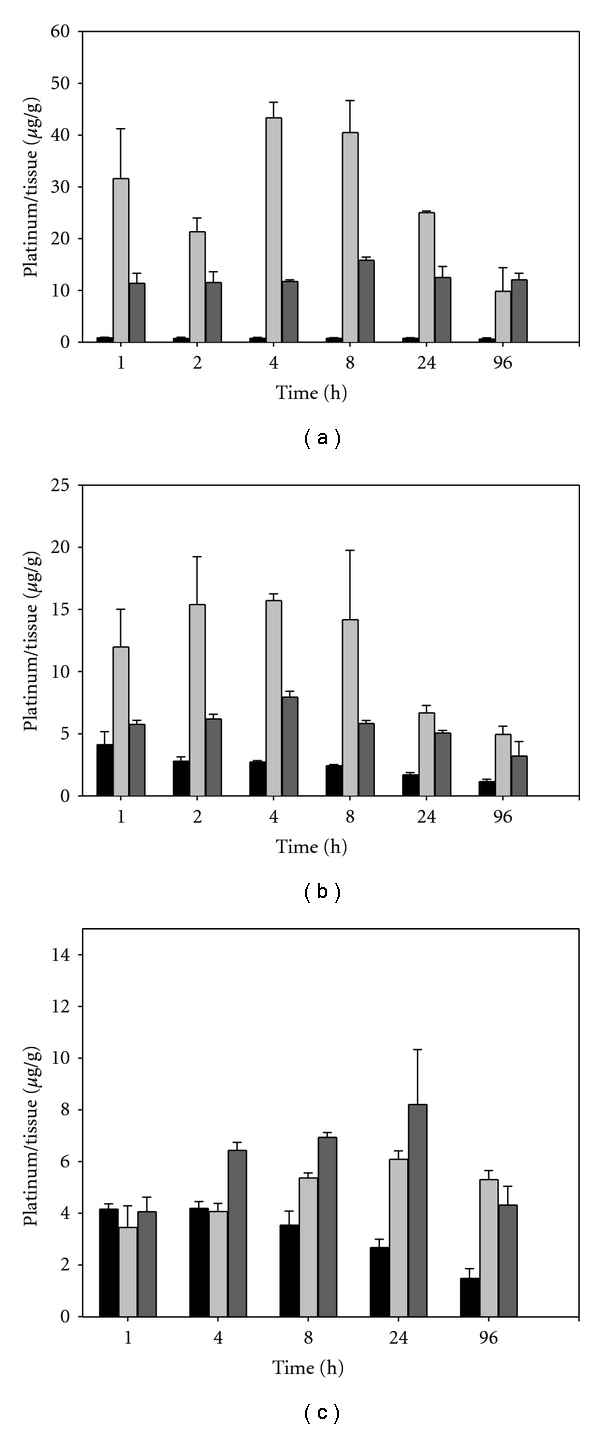
Accumulation of platinum in the spleen (a), liver (b), and kidneys (c) of BDF-1 mice (*n* = 3, Mean ± SD) following the injection of cisplatin at 3 mg/kg as free drug (■); liposomal cisplatin DSPC : DSPG : CHOL (7 : 2 : 1) (light-gray square), or liposomal cisplatin DSPC : DPPC : DSPG : CHOL (35 : 35; 20 : 10) (dark-gray square).

One of the major dose-limiting toxicities associated with the use of cisplatin is nephrotoxicity resulting from the accumulation of cisplatin within the kidney. To determine if the liposomal formulations of cisplatin had an impact on drug biodistribution, we compared the most and least therapeutically active formulations to free cisplatin at an equivalent drug dose. At various times after injection, kidney as well as the spleen and liver (established sites of liposomal accumulation) were harvested and assayed for platinum levels (Figure 4). Unfortunately, due to the reactivity of cisplatin, tissue extraction and analysis of intact drug by HPLC was not possible. As a result, we measured tissue levels of platinum using atomic adsorption spectroscopy as a marker for cisplatin content. Platinum accumulation in all 3 organs was higher following the injection of liposomal cisplatin compared to free cisplatin injection. The slow-releasing DSPC : DSPG : Chol (7 : 2 : 1) formulation had the highest levels of platinum in both the liver and spleen compared to the DSPC : DPPC : DSPG : Chol (35 : 35 : 20 : 10) formulation and free drug. Since the liver and spleen typically account for the majority of liposomal accumulation, it is not surprising that a slow-releasing formulation increased cisplatin delivery to these organs. In the case of the kidney, however, platinum levels were similar between both the formulations and the free drug. A slight increase in kidney levels over time in the blended formulation may represent an accumulation of free cisplatin released from the liposome as opposed to enhanced liposomal accumulation over time. Comparable kidney accumulation may account for the similarity in MTD doses of all three drug forms.

## 4. Conclusion

It is generally believed that increasing the retention of a drug within a delivery vehicle will result in enhanced drug accumulation at a tumor site. This was indeed shown to be true with SPI-077, where high levels of platinum were detected at tumor sites [16, 17]. Unfortunately this formulation showed little activity when evaluated clinically [12]. The limited therapeutic activity of that formulation matches well with the data presented in our paper where a formulation with high drug retention had the lowest therapeutic activity. Clearly, if cisplatin retention is so high that the release rate of the drug at the tumor site is below the therapeutic threshold, then no antitumor activity will be observed. Also, greater liposome retention can increase organ exposure and enhance toxicity, negating the protective effect of liposomal encapsulation. As the cisplatin release rate is enhanced, more free drug will be available to the tumor, resulting in cell killing and enhanced efficacy. For formulation that released cisplatin too quickly (T_1/2_ 4.5 hours) the opposite problem was observed. In the high %DP formulation, cisplatin release is occurring rapidly in the plasma which limits tumor exposure of the drug and limits the therapeutic effect. 

This paper describes the optimization of a liposomal carrier for enhanced cisplatin therapeutic activity. A liposomal formulation composed of DSPC : DPPC : DSPG : Chol (35 : 35 : 20 : 10) with an intermediate cisplatin half-life of 8.3 hours demonstrated the greatest efficacy in a P388 murine lymphocytic leukemia model. This optimized formulation has been utilized as part of CPX-571, a fixed-ratio drug combination of irinotecan and cisplatin. Superior antitumor activity has been reported for the liposomal irinotecan:cisplatin combination at a synergistic 7 : 1 molar ratio compared to free drug cocktail in all tumor models tested [22].

## Figures and Tables

**Table 1:** Correlation between membrane composition and pharmacokinetics parameters of cisplatin-containing liposomes.

Formulation	% DS	% DP	*T*_*m*_ (°C)	Cisplatin *T*_1/2_ (h)	AUMC cisplatin (mg·h^2^/mL)	Lipid *T*_1/2_ (h)	AUMC lipid (mg·h^2^/mL)
DSPC : CHOL (55 : 45)	55	0	55.3	18.9	82.6	23.5	12894.5
DSPC : DSPG : Chol (7 : 2 : 1)	90	0	54.9	14.7	27.8	28.0	9892.3
DSPC : DPPC : DSPG : Chol (6 : 1 : 2 : 1)	80	10	52.5	11.6	22.1	29.3	11836.8
DSPC : DPPC : DSPG : Chol (35 : 35 : 20 : 10)	55	35	48.8	8.3	6.6	15.3	2953.0
DSPC : DPPC : DPPG : Chol (35 : 35 : 20 : 10)	35	55	45.5	6.8	4.8	16.6	3511.0
DPPC : DPPG : Chol (7 : 2 : 1)	0	90	42.2	4.5	1.7	9.3	1058.9

The *T*_1/2_ and AUMC values presented in this table were based on plasma measurements recorded within the first 24 hours of administration.
